# Attitudes Toward the Use of Voice-Assisted Technologies Among People With Parkinson Disease: Findings From a Web-Based Survey

**DOI:** 10.2196/23006

**Published:** 2021-03-11

**Authors:** Orla Duffy, Jonathan Synnott, Roisin McNaney, Paola Brito Zambrano, W George Kernohan

**Affiliations:** 1 School of Health Sciences Faculty of Life and Health Sciences Ulster University Newtownabbey United Kingdom; 2 Institute of Nursing and Health Research Faculty of Life and Health Sciences Ulster University Newtownabbey United Kingdom; 3 School of Computing Faculty of Computing, Engineering and the Built Environment Ulster University Newtownabbey United Kingdom; 4 Department of Human Centered Computing Faculty of Information Technology Monash University Clayton Australia; 5 School of Computer Science, Electrical and Electronic Engineering and Engineering Maths Faculty of Engineering University of Bristol Bristol United Kingdom

**Keywords:** Parkinson disease, mobile phone, telerehabilitation, eHealth

## Abstract

**Background:**

Speech problems are common in people living with Parkinson disease (PD), limiting communication and ultimately affecting their quality of life. Voice-assisted technology in health and care settings has shown some potential in small-scale studies to address such problems, with a retrospective analysis of user reviews reporting anecdotal communication effects and promising usability features when using this technology for people with a range of disabilities. However, there is a need for research to establish users’ perspectives on the potential contribution of voice-assisted technology for people with PD.

**Objective:**

This study aims to explore the attitudes toward the use of voice-assisted technology for people with PD.

**Methods:**

A survey was approved for dissemination by a national charity, Parkinson’s UK, to be completed on the web by people living with the condition. The survey elicited respondent demographics, PD features, voice difficulties, digital skill capability, smart technology use, voice-assisted technology ownership and use, confidentiality, and privacy concerns. Data were analyzed using descriptive statistics and summative content analysis of free-text responses.

**Results:**

Of 290 participants, 79.0% (n=229) indicated that they or others had noticed changes in their speech or voice because of the symptoms of their condition. Digital skills and awareness were reported on 11 digital skills such as the ability to *find a website you have visited before*. Most participants (n=209, 72.1%) reported being able to perform at least 10 of these 11 tasks. Similarly, of 70.7% (n=205) participants who owned a voice-assisted device, most of them (166/205, 80.9%) used it regularly, with 31.3% (52/166) reporting that they used the technology specifically to address the needs associated with their PD. Of these 166 users, 54.8% (n=91) sometimes, rarely, or never had to repeat themselves when using the technology. When asked about speech changes since they started using it, 25% (27/108) of participants noticed having to repeat themselves less and 14.8% (16/108) perceived their speech to be clearer. Of the 290 respondents, 90.7% (n=263) were not concerned, or only slightly concerned, about privacy and confidentiality.

**Conclusions:**

Having been added to the homes of Western society, domestic voice assist devices are now available to assist those with communication problems. People with PD reported a high digital capability, albeit those who responded to a web-based survey. Most people have embraced voice-assisted technology, find it helpful and usable, and some have found benefit to their speech. Speech and language therapists may have a virtual ally that is already in the patient’s home to support future therapy provision.

## Introduction

### Background

Globally, there are more than 6 million people diagnosed with Parkinson disease, and it is currently the fastest growing neurological disease worldwide [1]. Early presentation includes tremor, stiffness or rigidity, slow movement, impaired balance, poor coordination, and speech problems [2]. Although it usually affects people aged >50 years, it can also affect younger people [2].

Problems with speech occur in 90% of people with Parkinson disease [3] at some point in their condition and include monotonous tone, reduced pitch and loudness, variable rate, imprecise consonant production, and an unclear *breathy* voice [4,5]. These speech symptoms are caused by issues with neuromuscular control over the speech mechanism that can be classified under the umbrella term of *dysarthria* [6]. People with Parkinson disease have an abnormal perception of loudness levels to guide the correct production of volume in their speech [7] so that an individual will feel that they are shouting when speaking at a normal level. Recalibration of the internal perception of volume and effort is one of the goals of speech and language therapy (SLT) [8]. The impact of speech problems is wide, affecting activities of daily living, mood, and self-identity [3].

Early SLT intervention is important to address communication issues [8], but only a little more than half of all people with Parkinson disease have contact with a therapist (52% in the United Kingdom [9]; 59% in Australia [10]). Given the extremely high rates of this population who experience voice changes or are dissatisfied with how they communicate [11], this rate of access to SLT is alarmingly low. Lee Silverman Voice Treatment (LSVT) is the gold standard approach provided by SLT for improving vocal loudness in people with Parkinson disease (which is often the primary concern). Despite its benefits, LSVT is resource intensive, requiring significant personal and professional time investment and self-directed motivation to practice largely repetitive exercises [12]. Often, the intensity and effort required to finish a program of LSVT outweigh the perceived benefits. People with Parkinson disease report that practicing on their own can be difficult; they feel self-conscious, overburdened, and doubtful about the effectiveness of carryover from therapy sessions to everyday situations [13]. The limited access to SLT and resource intensity of clinical services warrants exploration of alternative methods to support people with Parkinson disease to communicate effectively.

### Application of Technology

Technology can offer a range of opportunities to support people with Parkinson disease during this process of home-based practice by structuring activities, adding gamified elements to increase enjoyment, and providing positive reinforcement and feedback. For example, improved engagement and enjoyment in vocal loudness exercises conducted with a digital game was described by users [14]; an innovative crowd-sourcing approach was explored to provide real-time, human feedback on speech for people with Parkinson disease, who uploaded structured speech samples via an app [15]. Participants could then use a practice area in the app, based on feedback received, to direct their home-based practice (eg, focus on volume using a decibel meter, focus on pacing using a metronome). Further work showed promising results for the use of a head-worn wearable device (Google Glass) as a volume training tool at home and an assistance device in social settings with cues to increase volume [16]. The glasses displayed real-time feedback of volume using a thumbs up symbol for positive reinforcement when a preset target was achieved. When discussing Google Glass, people with Parkinson disease explicitly described the benefits of the voice interaction functionality to access technology. Even those with pronounced speech difficulties found success with voice interaction [17]. Although this work on technology-assisted SLT for people with Parkinson disease seems promising, it is only now emerging as an area of research, and studies to date only explore interventions with small numbers of participants. Through this work, we explored the opportunities for widely used, off-the-shelf voice-assisted technologies (VATs, which implement voice interaction) in supporting people with Parkinson disease.

*Voice assistants* are software agents installed in devices such as phones, computers, or tablets or on purpose-built speakers [18]. They are capable of interpreting human speech and, depending on the command they receive, can complete different tasks (eg, tell the time or the weather, send and read text messages, make phone calls, set alarms, play music, and control various connected devices) [18]. Currently, one in 5 homes in the United Kingdom owns a voice-assisted speaker, a figure that is predicted to rise significantly in the coming years [19]. As many as 53% of homes in the United States own one voice-assisted speaker [20]. As such, these VATs are growing in popularity and are becoming pervasive. The older population (aged ≥60 years) make up around 20% of smart speaker ownership, with almost 60% of these consumers using the device every day [21]. Amazon Alexa is the market leader across all age groups [21]. VAT offers hands-free access and naturalistic voice interaction, a beneficial means of interacting with the device for those with physical disabilities or lower levels of technology literacy [15]. As such, recent years have seen an emergence in research in the health and care space, which is exploring the role of VAT in supporting people within these demographics.

A living lab study was conducted with older adults aged between 64 and 89 years [22] to explore older people’s interactions with a voice assistant (Google Home) and several connected smart home devices. Participants were asked to perform several relevant activities (eg, ask for information, control lights, fans, and a television [TV]) and were interviewed about their experiences. The authors noted high levels of acceptance with the smart home technologies among older adults and, in particular, described the value they found using voice command as an input, describing how participants enjoyed interacting at their own pace, without being *judged* or *hurried*. Similarly, the design of adaptive systems, using voice interface technology for people with physical disability, can enable flexible use of smart homes [23]. The VAT system was *self-learning* and adapted to each user’s command preferences after being trained through a series of short sessions. This work shows promise in particular for participants with speech difficulties, as the system adapts to impaired speech patterns (eg, people with dysarthria taking more pauses between phrases).

Several studies have explicitly explored the opportunities of the leading VAT (Amazon Alexa) to support people with disabilities, largely focusing on analysis from public reviews (posted on the Amazon store). For example, 284 reviews were thematically analyzed from people discussing disability and found recurrent themes relating to feelings of empowerment, as well as reporting success from people with speech difficulties [24]. They concluded that although very promising, usability issues, such as unintended access to the technology from children and privacy concerns, can have serious implications for health applications in the home. They also recognized the need to consider disease state in technical skill development to reduce frustrations. Similarly, 346 Amazon reviews by people with cognitive, sensory, or physical disability were analyzed, finding high levels of acceptance among users, reports of users considering the device as a companion, and increased reported independence in the user [25]. The authors also explicitly discussed reviews from users with speech difficulties. A total of 13.6% (47/346) of the reviews were by someone with speech impairment, and 74% (23/31) of their comments were around positive experiences with the technology, indicating success with being understood by Amazon Alexa. Interestingly, 2.0% (7/346) of users mentioned specifically that it helped them *talk slowly, clearly, and loudly*, which is highly relevant to our work with Parkinson disease (PD), where this is often the main aim of SLT. Similar findings were found in a study of the challenges and opportunities for the internet of things (IoT) for people with Parkinson disease [26]. Approximately 50% of the participants had already used Amazon Alexa in their homes, and similar reports from a participant with speech difficulties described speaking in a slower, clearer voice to enhance his ability to interact with Amazon Alexa [26]. This effect is interesting and potentially significant for speech improvement, justifying further investigation.

In addition, the extent to which people with dysarthria (the motor speech disorder experienced by people with Parkinson disease) interacted with 3 specific VATs (Apple Siri, Google Assistant, and Amazon Alexa) was investigated [27]. They used the TORGO database [28], consisting of available recordings of people with dysarthria, and found 50% to 60% accuracy of phrase recognition. What was not controlled for in this study was how well the VATs worked in correlation with the degree of dysarthric speech (ie, it worked better with a moderate level vs severe dysarthria or was the presence of any dysarthria, even a mild one, a cause for issue). In addition, the speech samples were standardized in nature and recorded in laboratories and thus did not represent the naturalistic interactions with the VATs that one would carry out in everyday life. Finally, the abovementioned study did not account for disease-specific origins of the dysarthria, which could in themselves have different factors that account for the intelligibility levels in the speech samples.

In summary, there is some evidence that VATs are already beginning to improve the lives of older people and people with disabilities and clear potential for the technology to support people with speech impairments. Furthermore, VATs may even be unexpectedly acting as a prompt for improving the speech of some users [24-26]. However, these studies provide only anecdotal evidence, highlighting the need to conduct systematic research to explore if and how people with different levels of speech impairment engage with VATs.

### Study Aims

In this work, we investigate the opportunities for VAT to support SLT outcomes for people with Parkinson disease. We focused on exploring the ownership and acceptance of VATs among the Parkinson community and their usability for those with speech issues. In addition, to further explore possible barriers to the adoption of VAT to the wider Parkinson community, we also wanted to explore any privacy and security concerns that people with Parkinson disease might have surrounding these technologies. We aimed to answer 3 questions within the UK-based Parkinson community:

1. What is the level of basic digital skills?
2. What is the knowledge and experience of existing VAT?
3. What are the reported effects of VAT on speech and language?

In so doing, we aim to build a foundation of knowledge for further research, development, and implementation of VAT for people with Parkinson disease.

## Methods

### Survey Design

Using a descriptive, observational approach, we developed a survey using Qualtrics, a web-based platform. The survey was based on a review of the literature on Parkinson speech and voice difficulties and digital technology use [26,29,30]. It was pilot tested with six academic staff members at two UK universities. Following amendment, it was further piloted with 44 patient and public involvement (PPI) volunteers, accessed through Parkinson’s UK. Volunteers were sent a link for the survey that they completed on the web, and they were asked to provide feedback on the survey with any suggestions for improvement. The PPI feedback resulted in amendments to the flow of the survey, removal and addition of questions, improving clarity, and improving format.

The final version of the survey consisted of 31 questions in four sections (Multimedia Appendix 1).

**Figure 1:**
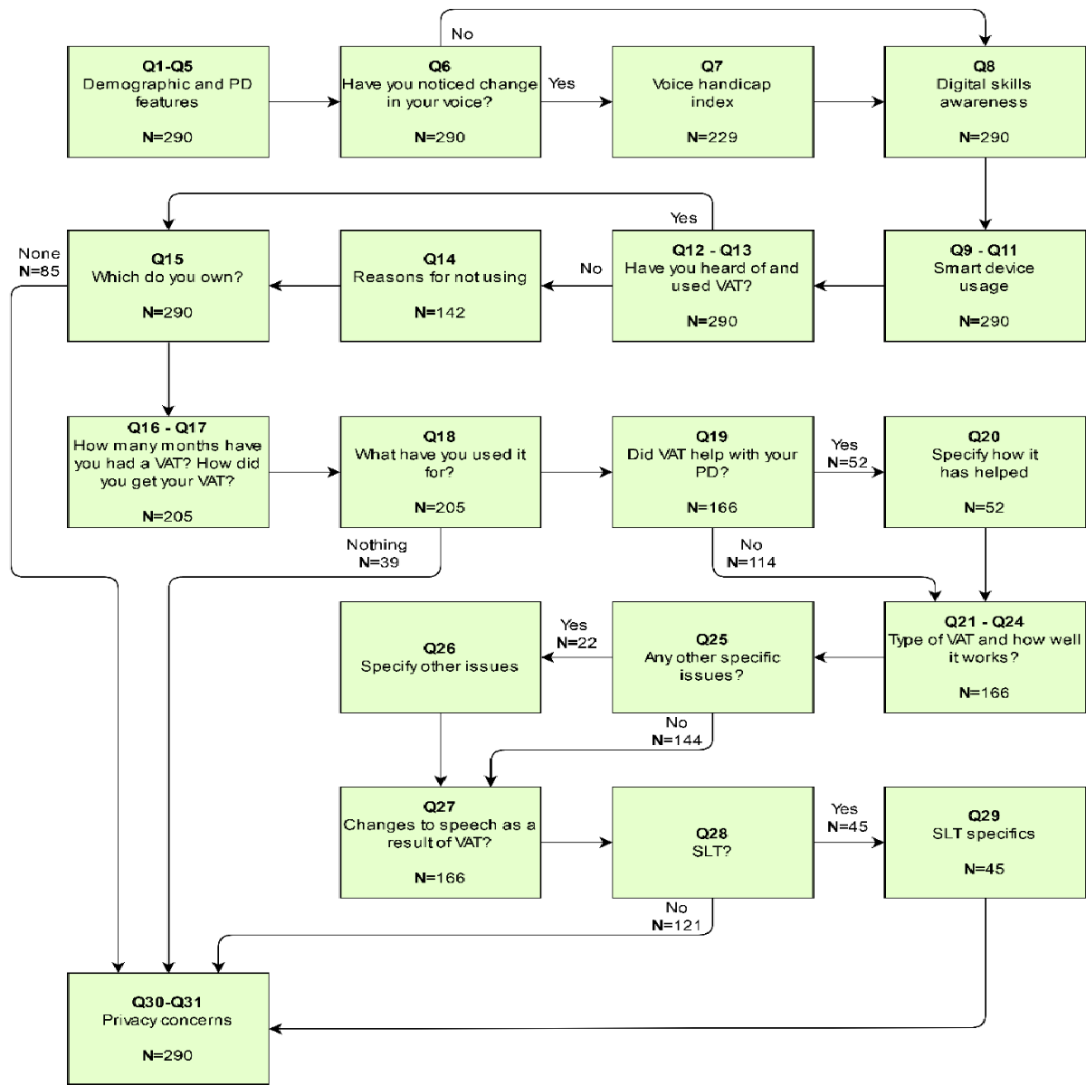
Survey flow diagram. Starting at top left, the diagram shows elements of the survey with skip logic to avoid unnecessary questions such as Voice Handicap Index, which applies to respondents who notice a change (Q7). Numbers of respondents to each element are given. The final element elicits security concerns (bottom left).

1. Six questions on demographics and PD features to elicit the profile of respondents.
2. Voice Handicap Index (VHI): we wanted to collect information on participants’ voice symptoms and their impact on their lives. As such, we used a validated instrument, the VHI [29]. The VHI is a voice outcome measure that has been used widely in voice research with various clinical and healthy populations and specifically with people with PD. It is validated with good psychometric properties. It assesses the physical, functional, and emotional impact of voice difficulties.
3. Digital skills and awareness: digital skills were assessed through an adaptation of the Tech Partnership’s Basic Digital Skills framework [30]. Reuse permission was also granted. This framework consists of asking respondents which of the 11 digital tasks they would be able to complete if they were asked. These digital skills cover areas including managing information, communicating, transforming, problem solving, and creating. For example, a Managing Information digital skill is Find a website I have visited before. This instrument collected details on digital ability.
4. Smart device usage: there were 3 questions about smart device usage, providing further details about digital access and familiarity. To find out about VAT specifically, there were 20 questions about usage, ownership, support for PD features, problems with usage, VAT impact on speech, and security concerns. Responses were in both free text and checkbox format. The survey used display logic to direct participants to relevant questions; therefore, different numbers of participants answered some questions (Figure 1).

### Study Population and Recruitment

Following peer review, the study was approved by the Institute of Nursing and Health Research Ethics Committee at Ulster University. Participants were presented with details about the study on the welcome page. Participants were made aware that all data were anonymous and would be used in the research. Informed consent was obtained by providing participants with information about the study, its purpose, length of time to complete, data storage, and anonymity. Consent was indicated through the submission of responses. Parkinson’s UK disseminated the survey by emailing it to their research support network from March 15 to April 30, 2019. We included people with Parkinson disease of any age and at any stage of the disease. The sample size was based on the number required to obtain 90% confidence and ±5% margin of error in estimating proportions: exact calculation, N=289.

### Analysis

The study used a mixed methods approach. Quantitative data were analyzed using the statistical package IBM-SPSS (version 26) [31]. Descriptive statistics, such as frequency, standard deviation, and mean, were used. Summative content analysis was used for qualitative free-text responses [32]. Responses from each free-text question were collated into a spreadsheet and separately analyzed by 2 researchers to identify themes. Any disagreements were resolved through discussion until a decision had been made on the final set of themes. Frequency counts were then provided, with the number of responses relating to each theme available for the analysis.

## Results

### Demographics and Digital Skills

The survey received responses from 320 respondents. Partially completed survey responses, which did not include completion of the final mandatory question, were excluded. This resulted in the exclusion of 30 respondents, providing a total of 290 fully completed surveys for analysis.

Of 290 respondents, 116 (40.0%) were female and 174 (60.0%) were male; the most represented age group was 65-74 years (121/290, 41.7%), followed by 55-64 years (96/290, 33.1%). Most respondents (237/290, 81.7%) were based in England, with 25 (8.6%) based in Scotland, 17 (5.9%) in Wales, and 11 (3.8%) in Northern Ireland. Respondents were asked to specify how many years it had been since their diagnosis. A total of 97.9% (284/290) of respondents had been diagnosed with PD for at least 1 year. The mean years since diagnosis was 6.35 (SD 5.55).

Respondents were asked to select which PD symptoms they experienced. Slow movement was the most commonly experienced symptom (227/290, 78.3%), followed by writing changes (223/290, 76.9%). Of particular relevance to this study was speech changes, which was the third most commonly reported symptom at 66.9% (194/290). In addition, 79.0% (229/290) of respondents indicated that they or others had noticed changes in their speech or voice because of their PD, and this group of 229 then were asked to complete the VHI. The VHI consists of 3 parts; each part provides 10 statements regarding speech difficulties and their impact on physical, functional, and emotional domains. Respondents were asked to respond to each statement with a score between 0 (never) and 4 (always), indicating how often they experienced each difficulty. Example statements are “My voice makes it difficult for people to hear me” and “My voice problem upsets me.” Higher scores indicate more severe vocal difficulties. Each section has a minimum possible score of 0 and a maximum possible score of 40. The minimum possible score for the entire VHI was 0, and the maximum possible score was 120. Table 1 provides an overview of the mean scores for each VHI section and for the overall VHI.

**Table 1:** Voice Handicap Index scores by domain.

Section		Score^a^
**Function**		
	Mean (SD)	16.06 (7.67)
	Range	37-0
**Physical**		
	Mean (SD)	16.09 (6.78)
	Range	34-0
**Emotion**		
	Mean (SD)	14.04 (8.70)
	Range	38-0
**Total**		
	Mean (SD)	46.19 (21.08)
	Range	108-1

^a^Voice Handicap Index scores: a higher score indicates more severe voice problems.

The scores for each statement were analyzed. In the Function section, the top-rated items were “People have difficulty understanding me in a noisy room” (mean 2.31, SD 0.94), “My voice makes it difficult for people to hear me” (mean 2.07, SD 0.78), and “People ask me to repeat myself when speaking face-to-face” (mean 2.00, SD 0.86). Of the 229 respondents, 3 scored 0 in this section. In the Physical section, the top-rated items were “The clarity of my voice is unpredictable” (mean 2.19, SD 0.91) and “The sound of my voice varies throughout the day” (mean 2.13, SD 0.89). In this section, 4 people scored 0, and they were not the same as the 3 respondents who scored 0 in the Function section. In the Emotions section, the top-rated item was “My voice problem upsets me” (mean 1.80, SD 1.18). In this section, 8 people scored 0; 2 of them scored 0 in part 2, and 1 scored 0 in part 1.

The Digital Skills questionnaire consisted of 11 items (eg, “use a search engine to look for information online”). Participants were asked to select a yes if they could complete the skill or no if they could not. Of the 290 participants who completed the questionnaire, 208 (71.7%) were able to complete at least 9 of 11 skills. The highest rated skill was “Use a search engine to look for information online”, as 97.6% (283/290) of respondents were able to complete it, closely followed by “Find a website you have visited before” (280/290, 96.6%) and “Send a personal message to another person via email or online messaging service” (275/290, 94.8%). “Create something new from existing online images, music or video” had the lowest number of participants indicating they would be able to complete it (132/290, 45.5%).

In summary, as many as 79.0% (229/290) of respondents indicated that they or others had noticed changes in their speech or voice because of PD. The respondents rated themselves as digitally competent, with 71.7% (208/290) being able to complete at least 9 of 11 digital skills.

### Smart Device Usage

Respondents (n=290) reported how familiar they were using technology, such as smartphones, computers, tablets, and laptops. More than half (163/290, 56.2%) of the respondents indicated that they were very familiar, 38.6% (112/290) indicated that they were somewhat familiar, and only 5.2% (15/290) indicated that they were unfamiliar with the use of these devices. Respondents (n=290) were asked how often they use technologies such as smartphones, computers, tablets, and laptops. The vast majority (272/290, 93.8%) of respondents indicated daily usage, 1.4% (4/290) indicated weekly usage, 1.7% (5/290) monthly, and 3.1% (9/290) indicated that they never used these devices. A total of 91.7% (266/290) respondents indicated that they own a touchscreen device, such as a smartphone or tablet.

The respondents were asked about the ownership of VAT. A total of 29.3% (85/290) participants said that they did not own a VAT and were directed to the last 2 questions of the survey, as the rest of the survey was concerned with ownership. The remaining 70.7% (205/290) participants responded to questions about how long they owned their device and how they had gained one. The respondents owned their VAT for a mean of 23 months (range 0-84 months, SD 18.5), with 70.2% (144/205) owning it for 24 months or less.

Of those who own VAT (n=205), 49.3% (101/205) bought it for themselves and 17.1% (35/205) received it as a gift and 2.9% (6/205) were recommended VAT by a health care professional. Other sources (63/205, 30.7%) included preinstallation on a smart device (47/63, 74.6%), provided for work or study access (8/63, 12.5%), and other general comments (8/63, 12.5%).

Respondents who owned VAT (n=205) were asked what they had used the technology to do. For this question, participants could select more than one response. The most popular responses were to request information (n=131, 64.0%), to play music (n=92, 44.9%), and to set a reminder (n=67, 32.7%). The *other* category was selected by 30.2% (n=62), and free-text responses included dictating messages and text (n=36/62, 58.1%), creating a shopping list (4/62, 6.5%), setting a timer (3/62, 4.8%), controlling the home environment (3/62, 4.8%), answering questions (3/62, 4.8%), and miscellaneous (13/62, 21.0%). Of those who owned a VAT, 19.0% (39/205) had not used it, and they were directed to the last two questions in the survey, as the remaining questions were about use. Therefore, 166 respondents answered the next set of questions.

### VAT for PD Support

A total of 166 respondents were asked if they had used VAT to help with their PD. A total of 31.3% (52/166) reported that voice assistants helped them with aspects of their PD. Most responses focused on using speech-to-text functions (33/52, 63.5%) to cope with symptoms such as *tremor, which makes typing difficult.* There were specific mentions about how VAT had helped respondents to practice their speech (7/52, 13.5%), for example, *“*Voice meter to practice voice levels*”* and *“*Low Volume speech. I have to concentrate to say ‘Alexa’ loud enough.” Other respondents used the technology to set medication reminders (4/52, 7.7%) to access entertainment, such as listening to music (3/52, 5.8%), and to communicate with other people through calls (4/52, 7.7%).

When queried about the type of VAT the 166 respondents used, 45.8% (n=76) used only mobile VAT, 30.1% (n=50) used only a standalone device, and 24.1% (n=40) used both.

### How Well Do Voice Assistants Work?

Table 2 provides an overview of how well voice assistants function for participants. Participants were asked how well the VAT works in general and specifically how well they feel the VAT understands their voice.

Participants were asked to explain their answers, with 86.7% (144/166) participants providing further explanation via a free-text box. A total of 36.1% (52/144) respondents of participants agreed that the device misinterpreted what they had asked, which could cause frustration; for example, “I find that it often misinterprets what I say so I spend a lot of time correcting it which is very frustrating.” More common problems related to PD and specifically with speech were also mentioned (19/144, 13.2%), such as “Sometimes Amazon Alexa does not hear me—because of my Speech problem with Parkinson’s”; “Due to stumbling over words, or stuttering, or low gravelly voice misunderstands me”; “Sometimes my voice is too quiet for Apple Siri.” Several participants noted that intonation or accent (9/144, 6.3%) affected this technology; for example, “I have a Scottish accent and so some voice technology does not understand my accent.” Other responses were related to the fact that participants did not use the technology frequently (13/144, 9%), that they were in an early *training phase* of using the technology (7/144, 4.9%), or that there were general technical issues (6/144, 4.2%).

Of the 166 respondents, 12.0% (20) who had used VAT reported other specific issues while using VAT. Misinterpretation of what participants had said was one of the main issues reported (6/20, 30.0%). For example, *misunderstood words and proper nouns*. Grammar was also cited as a problem for 15% (3/20) of participants, for example, “Always inserts capital letter. Correcting it is not easy.” In addition, there were (6/20, 30.0%) responses that specifically discussed technical restrictions of the technology itself and how this could cause issues. Some of the participants (3/20, 15.0%), however, highlighted that some positive speaking behaviors might arise through issues with the technology, for example, *have to speak slowly and clearly*; *having to talk louder*; and *making my voice clear.* Another (1/20, 5.0%) reported concerns over the privacy of their personal information.

**Table 2:** How well voice-assisted technology works for participants and how well it elicits meaning in their speech (N=166).

How well VAT^a^ works			Values, n (%)
**How well does the VAT work for you?**			
	It always works for me	20 (12.0)	
	It works most of the time	72 (43.4)	
	It works about half of the time	17 (10.2)	
	It works some of the time	52 (31.3)	
	It never works for me	5 (3.0)	
**How well do you feel the VAT understands your voice?**			
	I never have to repeat myself	7 (4.2)	
	I rarely have to repeat myself	23 (13.9)	
	I sometimes have to repeat myself, but it works most of the time	61 (36.7)	
	OK, but I often have to repeat myself	44 (26.5)	
	I usually have to repeat myself	16 (9.6)	
	I always have to repeat myself	15 (9.0)	

^a^VAT: voice-assisted technology.

### Speech Changes as a Result of VAT

Respondents with PD were asked about changes in their speech as a result of using VAT. Table 3 provides an overview of responses from 166 who use VAT and from people with Parkinson disease who recorded speech changes as a symptom they experience. The most common response was “I have not noticed any change in my speech” (87/166, 52.4% overall; 71/166, 42.6% of people with Parkinson disease with speech changes), and the least common response was “Confidence in my speech has decreased” (11/166, 6.6% overall; 15/166, 9.3% of people with Parkinson disease with speech changes). As many as 25.3% (42/166) of participants who had identified speech changes reported that VAT asks them to repeat less.

**Table 3:** Changes to speech as a result of using voice-assisted technology by the overall population and by respondents who experience speech changes as a symptom of Parkinson disease.

Changes to your speech as a result of using your voice-assisted technology (percent that agree or strongly agree)	Overall (N=166), n (%)	With symptom: speech changes (n=108), n (%)
I have not noticed any change in my speech	87 (52.4)	46 (42.6)
The voice assistant asks me to repeat myself less than when I first started using the technology	39 (23.5)	27 (25.0)
I feel my voice is clearer	24 (14.5)	16 (14.8)
I feel my voice is louder	20 (12.0)	14 (13.0)
Confidence in my speech has increased	18 (10.8)	15 (13.9)
Other people ask me to repeat myself less than when I first started using the technology	14 (8.4)	11 (10.2)
Confidence in my speech has decreased	11 (6.6)	10 (9.3)

### Privacy and Confidentiality Issues

All 290 participants responded to questions relating to privacy and confidentiality issues associated with the use of VAT. A minority of respondents (27/290, 9.3%) were very concerned, 34.5% (100/290) were slightly concerned, and 56.2% (163/290) were not concerned at all. Respondents who did have privacy and confidentiality concerns were invited to provide further information about these concerns, with 30.0% (87/290) responding in free text. Of these, the biggest concern from participants was related to the possibility that they could be *hacked* (23/87, 26%), such as “being spied on and hackers”. The second most discussed concern was related to the storage and misuse of personal data (20/87, 23%), for example, “the surveillance potential in these devices is alarming. Information could be used to my detriment—health insurance, for example.” Another theme that was widely commented on was the fact that devices were *always listening* and how this might be used for surveillance purposes (13/87, 15%): “If the voice control technology is permanently active then you have a ‘Big Brother’ situation.” Finally, there were general comments regarding privacy (9/87, 10%), for example, “TV documentaries have shown that Amazon can collect information on users of Amazon Alexa, so they have no privacy”; security (8/87, 9%); and confidentiality concerns (4/87, 5%).

In summary, 71% (205/290) of participants owned VAT, and 166 of them used their VAT device; of these 290 participants, 52 (31%) reported using VAT to help with their PD. Of the 166 participants, 54.8% (91) never or only sometimes had to repeat themselves when using VAT. When asked about speech changes since using VAT, as many as 25.0% (27/108) noticed that VAT asked them to repeat less when compared with when they started using it, and 14.8% (16/108) noticed that their speech was clearer. Of the 290 respondents, 90.7% (n=263) were not concerned or only slightly concerned about privacy and confidentiality.

## Discussion

### Principal Findings

The purpose of this study was to understand the attitudes and experiences of people with Parkinson disease toward VAT and to investigate their digital capabilities. Specifically, we were interested in any reported changes to speech and language through the use of VAT. We found that most respondents reported changes in their speech or voice because of PD, with almost 80% indicating this symptom. Interestingly, a large proportion (71%) of participants owned VAT, with almost one-third using VAT to help with PD symptoms. Of particular interest is that one-fourth of participants using VAT reported that it asks them to repeat less since they started using it.

The participants in this study could be considered representative of people with Parkinson disease, in agreement with other studies, by the proportion experiencing speech changes and the nature of their symptoms [11]. On average, they reported a moderate voice impairment as measured by the VHI [29], emphasizing the impact on the quality of life and supporting the need to explore solutions. The top-rated items in the VHI indicate issues with volume, clarity, and predictability of voice, all of which may be a challenge when communicating with VAT; however, recent studies have found that participants report putting in extra effort to optimize their speech when interacting with the device [24,26]. Future research is needed to fully explore the impact of VAT usage on speech in PD.

This survey explored digital skills and capabilities and found that most respondents were capable of completing most of the basic digital skills. The task that the least respondents indicated they could complete was creating something new from existing web-based images, music, or video. Nevertheless, a task of this nature is beyond the complexity of the VAT interaction. Overall, this level of basic digital skills, technology familiarity, usage, and ownership indicate a community in which the majority are actively embracing technology. Similarly, high rates of ownership and adoption of technology with older adults were found in a recent study [33]. This is a welcome result for technology developers, as this ultimately reduces the barriers to uptake of novel solutions for the Parkinson community, which our findings have indicated as digitally capable.

The results of this survey provide a positive outlook toward the knowledge and experience of existing VAT, with a high level of ownership and usage, showing a readiness to engage with new technology. Similar findings were found in a study exploring the IoT for support in people with Parkinson disease [26]. The accessibility features of voice activation for individuals who may be experiencing manual dexterity difficulties could contribute to this positive attitude among people with Parkinson disease [26]. However, we need to be cautious in our interpretation, as this self-selecting group may have responded because of their familiarity with VAT.

Respondents were asked how they obtained their VAT. A small proportion (2.9%) of respondents indicated that they were recommended by a health care professional, suggesting that an increased evidence base with regard to the potential benefits of VAT for people with Parkinson disease, combined with closer communication with health care professionals, may be required. Future research should consider the current knowledge and experience of speech and language therapists using VAT.

Almost one-third of our participants used VAT to help with their PD symptoms. A small number of studies reported that VAT helped them to successfully practice their speech by concentrating on increasing their volume or clarity as to be understood by the device, which is similar to findings from other recent papers [24-26]. It is important to recognize that there may be a misconception that VAT is not an option for people with Parkinson disease who experience speech changes, yet VAT offers some participants the encouragement to speak slower and louder. Perhaps, the opportunity for unlimited attempts, with clear indicators of success and the absence of frustration from a communication partner, makes this technology an attractive option. Such preliminary positive findings indicate the need for further research into how VAT can work for people with speech difficulties, as well as support and perhaps improve speech difficulties in people with Parkinson disease.

Participants using VAT were asked about speech changes as a result of using this technology. One-fourth of the respondents experiencing speech changes noted that the VAT asked them to repeat themselves less than when they began using the technology. This suggests that out-of-the-box VAT use may actually improve speech. Although we need to be cautious in this interpretation, it is possible that there are other reasons for being asked to repeat less: increased familiarity with the technology, increased awareness of the most reliable voice commands, and the VAT can improve voice recognition rather than speech improvement. This finding warrants further research.

One note of caution is that a few respondents with speech changes indicated a decrease in confidence in their speech since using VAT. It is not certain that this decline in confidence is a direct result of VAT rather than a progression of PD. However, this result highlights the possibility that repeated unsuccessful engagement with VAT may be detrimental to confidence and that use by people with Parkinson disease should be monitored, particularly in the early stages of use. Coyne et al [24] found that users were frustrated when VAT did not understand their voice because of speech impairments. Future work should ensure that speech recognition can be as accurate as possible for individual speakers with speech impairments to ensure that their confidence is strengthened and not eroded.

Nevertheless, a notable portion of respondents with speech changes indicated that they felt their voice was clearer (16/108, 14.8%) and louder (14/108, 13.0%), that their confidence in speech had increased (15/108, 13.9%), and that other people ask them to repeat themselves less than when they started using the technology (11/108, 10.2%). Although these changes were noticed by a minority of respondents, they do provide some promise that VAT may provide therapeutic benefit to people with Parkinson disease. It is interesting to consider the possibility that speech improvements reported by participants were experienced beyond the voice interaction with VAT. It is widely recognized that there are problems for people with Parkinson disease with maintenance and generalization of speech improvements from therapy tasks into everyday contexts [34]. The potential for VAT to improve social participation warrants further investigation.

Privacy and confidentiality concerns with VAT are a current topic of significant discussion within the media and academia [35]. The results from our survey indicate that the majority of respondents were not seriously concerned about privacy and confidentiality. However, more than one-third of respondents did have slight concerns, and 9.3% were very concerned. Specific concerns were around the potential for hacking, misuse of personal data, and surveillance potential. This prevalence of concern is similar to that found in another study, in which 7% of participants reported privacy concerns as a reason for not using such devices and concluded that these concerns influence the likelihood of using the device and trust in commercial companies [36]. Interestingly, research has found that individuals might be more likely to share data for the benefit of their care and others [26,37]. To maximize the uptake of these technologies and benefit from the immense potential offered to patients’ health, further efforts must be made to reassure, promote clear privacy-friendly default settings in companies, and educate potential users about privacy and confidentiality concerns.

### Limitations

The survey was advertised and distributed electronically. Therefore, it is logical to assume that respondents primarily consisted of self-selecting people with Parkinson disease who are already actively engaging with technology. Nevertheless, this method of distribution facilitated the collection of a higher number of responses than would have otherwise been possible. Another potential limitation of this study is that it is possible that people with Parkinson disease who are familiar with VAT may have been more likely to engage with the survey than those who have no experience with VAT. This is a limitation of any survey that focuses on a particular subject.

### Conclusions

Many people with Parkinson disease recognize that they are experiencing voice and speech changes because of their condition. This group of participants reported some promising effects on their speech symptoms when using VAT; however, this needs further investigation. This is the first study to systematically explore the experiences of using VAT by people with speech difficulties. The next step will be to investigate speech and language therapists’ current professional use of VAT and to consider their professional opinion of VAT as a potentially useful support for speech improvement. More research is needed to trial out-of-the-box VAT for speech and communication difficulties in people with Parkinson disease and explores the potential generalization effects that might occur in other nontechnology-mediated speaking contexts.

## Appendix

Multimedia Appendix 1 Full survey.

## Abbreviations

IoT: internet of things
LSVT: Lee Silverman voice treatment
PD: Parkinson disease
PPI: patient and public involvement
SLT: speech and language therapy
TV: television
VAT: voice-assisted technology
VHI: Voice Handicap Index
