# Supplementary material for: Attitudes Toward the Use of Voice-Assisted Technologies Among People With Parkinson Disease: Findings From a Web-Based Survey
Source: JMIR Rehabil Assist Technol. 2021 Mar 11;8(1):e23006. doi: 10.2196/23006 (PMC8082949; doi:10.2196/23006)
Supplement: Multimedia Appendix 1 [file rehab_v8i1e23006_app1.pdf]

Multimedia Appendix

This is a Multimedia Appendix to a full manuscript published in the J Med Internet Res. For full copyright and citation information see

Full survey

Understanding Attitudes towards Voice Assisted Technologies by people affected by Parkinson's

Survey Introduction

Hello. Thanks for opening the survey.

We are interested in what you think about voice assisted technology. Voice assisted technologies, such as Alexa and Siri, mean that you can request information, organise schedules and control entertainment with your voice. Your responses will ensure that views of people affected by Parkinson's are heard; they will have an influence on future work in this area. This survey is a collaboration between Ulster and Bristol Universities.

Instructions If you are willing to take part, please read the information sheets attached and then complete the questions. The survey should take between 10 and 20 minutes depending on how much you want to add. Please note that you are able to withdraw from the survey if you have not submitted your responses. We will discount any surveys that are not completed. Once you have submitted your answers to this survey they cannot be withdrawn. Your responses are anonymous so therefore we cannot find your specific responses once they are submitted. We have specific duties to look after your data and manage in accordance with good practice and the law. In brief, we plan to secure your data safely and make anonymised datasets available for other researchers. Please see attached the GDPR privacy statement and follow the link to the data management plan https://www.ulster.ac.uk/about/governance/compliance/gdpr. Please take a moment to look at them. If you become worried or upset by anything in this survey please follow the link to Parkinson’s UK who are experienced in helping with your condition. https://www.parkinsons.org.uk/. Alternatively please make contact with your GP. The survey uses two published questionnaires. 1. The Voice Handicap Index (VHI): Development and Validation. Barbara H.Jacobson, Alex Johnson, Cynthia Grywalski, Alicew Silbergleit, Gary Jaconsen, Michael S. Benninger. American Journal of Speech-Language Pathology, Vol 6(3), 66-70, 1997 2. The Tech Partnership Get Digital Basic Skills Assessment Questions. Re-use permission granted.

Q1 Section 1- About You

What age are you?

- ◯ Under 35 (1)
- ◯ 35-44 (2)
- ◯ 45-54 (3)
- ◯ 55-64 (4)
- ◯ 65-74 (5)
- ◯ 75-84 (6)
- ◯ 85+ (7)

Q2 Are you male or female?

- ◯ Female (1)
- ◯ Male (2)

Q3 Where do you live?

- ◯ England (1)
- ◯ Northern Ireland (2)
- ◯ Scotland (3)
- ◯ Wales (4)

Q4 How many years has it been since you were diagnosed with Parkinson's?

____

Q5 What are the main Parkinson's-related symptoms that you currently experience? (Tick all that apply).

- ▢ Tremor (4)
- ▢ Slowed movement (5)
- ▢ Rigid muscles (6)
- ▢ Impaired posture and balance (7)
- ▢ Loss of automatic movements (8)
- ▢ Speech changes (9)
- ▢ Writing changes (10)
- ▢ Other (please describe below) (11)

____

Q6 Have you or others noticed any changes in your speech or voice due to your Parkinson's?

- ○ Yes (1)
- ○ No (2)

Skip To: Q11 If Have you or others noticed any changes in your speech or voice due to your Parkinson's? = No

Q7 Section 2- Voice Handicap Index

These are statements that many people have used to describe their voices and effects of their voices on their lives. Mark the response that indicates how often you have the same experience. Please see Barbara H.Jacobson, Alex Johnson, Cynthia Grywalski, Alicew Silbergleit, Gary Jaconsen, Michael S. Benninger. American Journal of Speech-Language Pathology, Vol 6(3), 66-70, 1997

Part I- Function
Part II- Physical
Part III- Emotion

Q8 Section 3- Digital Skills and Awareness

Please complete the following table to help us understand your level of digital skills.

	I can do this	
	Yes (1)	No (2)
Use a search engine to look for information online (1)	○	○
Download/save a photo you found online (2)	○	○
Find a website you have visited before (3)	○	○
Send a personal message to another person via email or online messaging service (4)	○	○
Make comments and share information online (5)	○	○
Buy items or services from a website (6)	○	○
Buy and install apps on a device (7)	○	○
Solve a problem you have with a device or digital service using online help (8)	○	○
Verify sources of information you found online (9)	○	○
Complete online application forms which include personal details (10)	○	○
Create something new from existing online images, music or video (11)	○	○

Q9 How familiar are you with using technology such as smartphones, computers, tablets and laptops?

- ○ Very familiar (1)
- ○ Somewhat familar (2)
- ○ Unfamiliar (3)

Q10 How often do you use technology such as smartphones, computers, tablets and laptops?

- ○ Daily (1)
- ○ Weekly (2)
- ○ Monthly (3)
- ○ Never (4)

Q11 Do you own a touch screen device? (A smartphone or tablet)

- ○ Yes (1)
- ○ No (2)

Q12
Section 4- Voice Assisted Technology and Parkinson's

Which of the following voice assisted technologies have you heard of? (Tick all that apply).

- ☐ Alexa (1)
- ☐ Siri (2)
- ☐ Cortana (3)
- ☐ Google Assistant (4)
- ☐ Other (please specify) (5) ____
- ☐ ⊗None of these (8)

Q13 Have you used a voice assisted technology in the past?

- ○ Yes (1)
- ○ No, but I would like to use it (2)
- ○ No, and I would never use it (3)

Skip To: Q20 If Have you used a voice assisted technology in the past? = Yes

---

Q14 Is there a particular reason why you have not used voice assisted technologies? (Tick all that apply)

- ☐ I'm not interested in using technology (1)
- ☐ Technology is too expensive (2)
- ☐ Technology is too complicated (3)
- ☐ I don't know what technology is available (4)
- ☐ I have not had any training (5)
- ☐ I have never had the opportunity to use it (6)
- ☐ Other (please explain below) (7)

____

Q15 Which, if any, of the following voice assisted technologies do you own? (Tick all that apply)

- ☐ Alexa (1)
- ☐ Siri (2)
- ☐ Cortana (3)
- ☐ Google Assistant (4)
- ☐ Other (please specify) (7)

____

- ☐ ⊗Don't own any (11)

Skip To: Q35 If Which, if any, of the following voice assisted technologies do you own? (Tick all that apply) = Don't own any

---

*

Q16 How many months have you had a voice assisted technology?

____

Q17 How did you get your Voice Assisted Technology?

○ I bought it for myself (1)

○ I received it as a gift (2)

○ It was recommended by a health care professional (4)

○ Other(Please specify) (5) ____

Q18 What have you used the voice assisted technology to do? (Tick all that apply)

☐ To play music (1)

☐ To request Information (2)

☐ To set a reminder (11)

☐ Other (Please specify) (12) ____

☐ ⊗I haven't used it (13)

Skip To: Q35 If What have you used the voice assisted technology to do? (Tick all that apply) = I haven't used it

Q19 Have you used voice assisted technology to help with your Parkinson's?

- ○ Yes (1)
- ○ No (4)

Skip To: Q26 If Have you used voice assisted technology to help with your Parkinson's? = No

Q20 What aspects of your Parkinson's have you used it to help with?

____

____

____

____

____

Q21 What type of voice assisted technology do you use? (Tick all that apply)

- ▢ Mobile phone voice assisted technology such as Siri (1)
- ▢ Standalone voice assisted technology such as Alexa (2)

Q22 How well does the voice assisted technology work for you? (Select one)

- ○ It never works for me (1)
- ○ It works some of the time (2)
- ○ It works about half of the time (3)
- ○ It works most of the time (4)
- ○ It always works for me (5)

Q23 Please explain you answer.

____

____

____

____

____

Q24 How well do you feel the voice assisted technology understands your voice? (Select one).

- ◯ I always have to repeat myself more than once (1)
- ◯ I usually have to repeat myself (2)
- ◯ OK, but I often have to repeat myself (3)
- ◯ I sometimes have to repeat myself, but it works most of the time (4)
- ◯ I rarely have to repeat myself (5)
- ◯ I never have to repeat myself (6)

Q25 Have you had any other specific issues with using the voice assisted technology?

- ◯ Yes (1)
- ◯ No (2)

Skip To: Q32 If Have you had any other specific issues with using the voice assisted technology? = No

Q26 What issues have you had with using voice assisted technology?

____

Q27 Please indicate your level of agreement to the following statements regarding changes to your speech as a result of using your voice assisted technology.

	0-Strongly Disagree (1)	1-Disagree (2)	2-Maybe, but I'm not sure (3)	3-Agree (4)	4-Strongly Agree (5)
I feel my voice is louder (1)	○	○	○	○	○
I feel my voice is clearer (2)	○	○	○	○	○
The voice assistant asks me to repeat myself less than when I first started using the technology (3)	○	○	○	○	○
Other people ask me to repeat myself less than when i first started using the technology (4)	○	○	○	○	○
Confidence in my speech has increased (5)	○	○	○	○	○
Confidence in my speech has decreased (6)	○	○	○	○	○
I have not noticed any changes in my speech (7)	○	○	○	○	○

Q28 Have you been having speech and language therapy in the time that you have been using your voice assisted device?

- ◯ Yes (1)
- ◯ No (2)

Skip To: Q35 If Have you been having speech and language therapy in the time that you have been using your voice... = No

Q29 Please provide more information about the speech and language therapy that you have been having.

____

____

____

____

____

Q30 Do you have any concerns regarding privacy and confidentiality associated with the use of voice assisted technologies?

- ◯ Very concerned (1)
- ◯ Slightly concerned (2)
- ◯ Not concerned (3)

Q31 If you have any concerns, please describe below:

____

____

End of Block: Default Question Block
